# Photo-induced manipulation and relaxation dynamics of Weyl-semimetals

**DOI:** 10.1038/s41524-025-01708-0

**Published:** 2025-07-07

**Authors:** Jakub Šebesta, Oscar Grånäs

**Affiliations:** 1 Division of Materials Theory, Department of Physics and Astronomy, Uppsala University, Box 516, Uppsala, 751 20 Sweden; 2 IT4Innovations, VSB - Technical University of Ostrava, 17. listopadu 2172/15, Ostrava-Poruba, 708 00 Czech Republic

**Keywords:** Electronic structure, Ultrafast photonics, Electronic properties and materials

## Abstract

The use of ultrashort laser pulses to manipulate properties or investigate a materials response on femtosecond time-scales enables detailed tracking of charge, spin, and lattice degrees of freedom. When pushing the limits of experimental resolution, connection to theoretical modeling becomes increasingly important to infer causality relations. Weyl-semimetals are a particular class of materials of recent focus due to the topological protection of the Weyl-nodes, resulting in a number of fundamentally interesting phenomena. This work provides a first-principles framework based on time-dependent density-functional theory for tracking the distribution of Weyl-nodes in the Brillouin-zone following an excitation by a laser pulse. Investigating the prototype material TaAs, we show that residual shifts in the Weyl-Nodes’ position and energy distribution are induced by a photo-excitation within femto-seconds through band-structure renormalization. Further, we provide an analysis of the relaxation pathway of the photoexcited band-structure through lattice vibrations.

## Introduction

Materials where the electronic band structure exhibits non-trivial topological states have garnered significant interest in recent years. In particular, semi-metals with band structures forming Weyl nodes (WNs) display remarkable physical phenomena such as negative magnetoresistance, anomalous Hall effect, non-local transport, and quantum oscillations in magnetotransport^1–3^. Additionally, the topological properties of these materials offer promising avenues for energy-efficient information storage and manipulation^4^. The topological protection in Weyl semi-metals is manifested through pairs of topologically protected band crossings near high symmetry lines in the bulk material^1–3,5–7^. These crossings form Weyl cones, which touch at the WNs^8,9^, and host Weyl quasiparticles with different chirality. The quasiparticles represent vortices of the Berry phase^5,10^, characterized by a non-vanishing topological invariant known as the Chern number *C*^1^. The stability of WNs is tightly connected to the source and drain properties of the Berry curvature, with annihilation occurring only by merging Weyl points of opposite Chern numbers. Manipulation of topological states is typically achieved through modulation of lattice degrees of freedom via THz radiation^4,11^, optical pumping^12,13^, or a combination of nano-structuring and external pumping^14,15^.

From a modeling perspective, significant conceptual work has been performed using low-energy models^1^. However, under strong pumping, the quasi-particle band structure may change significantly^16^, challenging the validity of these models to make quantitative predictions. Therefore, a full band structure description is necessary for direct comparison with experimental data in pump-probe scenarios, paving a way to a quantitative understanding and enabling the implementation of optimal control. Previous work by Shin et al. tracked anomalous conductivity features using the velocity field, employing real-time time-dependent density functional theory (RT-TDDFT) in a pseudo-potential framework^17,18^. Recent work also shows that response functions calculated on the basis of all-electron RT-TDDFT electronic structures provide an excellent avenue for direct comparison to experimental data^19^.

The position of the WNs in the Brillouin zone is determined by the quasi-particle band-structure. Hence, band-structure renormalizations and modifications induced by an applied laser pulse are of the essence to include when determining the time-dependent positions of the WNs. A promising route is to use the (generalized) TD-DFT approach^20–23^, typically used to determine time-dependent integral quantities such as the density of states (DOS) or magnetization. In this work, we exploited a theoretical framework to investigate the impact of strong electromagnetic fields on WN dynamics in a material-realistic setting. We separated the impact of strong fields from the relaxation of the pumped state through lattice motion and provided a framework for investigating response functions to deduce changes in the material’s general susceptibility. Our developments focus on the all-electron implementation of RT-TDDFT in the *Elk* code^24^.

## Results and discussion

### Ground state electronic structure

**Fig. 1:**
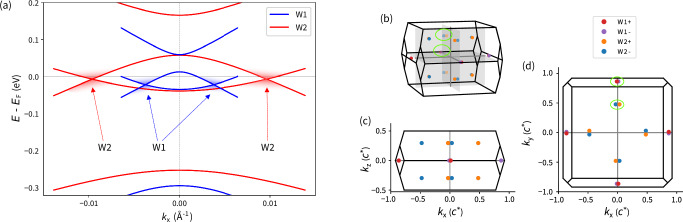
Weyl node position in the TaAs Brillouin zone. **a** Ground state Weyl nodes in TaAs. Band structure near the (blue) W1 and (red) W2 Weyl nodes along the lines connecting Weyl node pairs. **b**–**d** Positions of Weyl nodes within the BZ at the ground state.

**Fig. 2:**
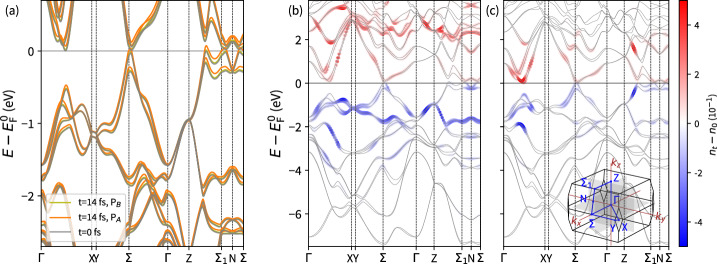
Pulse-induced band structure renormalization and change of band occupations. **a** Band structure renormalization induced by 3.9 eV P_A_ and 2.0 eV P_B_ laser pulses. Bands are depicted with respect to the ground state Fermi level ${E}_{{{\rm{F}}}}^{0}$. **b**, **c** Change of the band occupation after 14 fs. (**b**) P_A_ laser pulse (**c**) P_B_ laser pulse. Time-evolved band structures and related changes in band occupancy with respect to the ground state are depicted. The energy is scaled to the ground state Fermi level ${E}_{{{\rm{F}}}}^{0}$.

We employ our methods to study the impact of a laser pulse on TaAs, a well-known prototype of Weyl semi-metals. In order to demonstrate the capability to identify Weyl nodes (WNs) and determine their positions within the Brillouin Zone (BZ), ground state calculations without an applied external laser field were performed. These also served as the initial state of the TD-DFT propagation scheme used later to study the laser-induced dynamics. The obtained ground state electronic band structure (Figs. 1a and 2a) corresponds well with the literature^8^. For the selected k-path, the valence and conduction bands nearly touch at the Fermi level *E*_*F*_, between the *Σ*_1_, N, and *Σ* points, likely indicating the presence of WNs. Except for these regions, the conduction and valence bands remain gapped, consistent with the semi-metallic character of TaAs^9^.

After evaluating the band structure, we determined the precise location of the WNs employing the Wilson loop technique^25^ (Equations (18) and (19)) on an auxiliary adaptive *k*-point mesh, where the Wilson loop size was decreased by increasing the *k*-point sampling density in the vicinity of a Weyl node. The position as well as the chirality is derived from the sign of the integrated phase singularity along the Wilson loops (Figure SI3). Consistent with the literature, we observed two sets of WNs (Fig. 1). The first set comprises four pairs of W1 WNs lying in the *k*_x_*k*_y_-plane. These can be attributed to nearly touching bands at the Fermi level *E*_F_ near the *Σ* point (Fig. 2a). The second set includes eight pairs of W2 WNs with non-zero *k*_*z*_ components, indicated by band proximity near the *Σ*_1_ point. The WN mutual chiralities (Fig. 1) are depicted based on the relative topological charge. The W1 and W2 nodes differ not only by their *k*_z_ component but also by the separation of WN pairs in the *k*_x_*k*_y_-plane. The nodes in the W1 pairs are about twice as close as the W2 ones (Fig. 1)^9,26^. Additionally, the W1 nodes lie approximately 14 meV below the W2 nodes in energy, consistent with the literature^8,26^. This suggests that the W2 nodes are likely more significant for magneto-transport chiral anomalies^26^.

### Laser-induced dynamics and relaxation effects

Having verified the ground state positions of the WNs, we focused on the impact of an ultrafast laser pulse on the behavior of the WNs. Where a particular focus was put on their presence and modification relative to the ground state as a function of increasing time delay from the pump, including the relaxation pathways. To study a possible relaxation process following the laser pulse, a short pulse width of FWHM ~ 3.6 fs was selected for numerical stability and computational feasibility. To achieve a quantitative scaling of the system response, two distinct pulse strengths were considered (Methods section).

As anticipated, the applied laser pulse induced modifications in band occupancies due to electron state excitations (Fig. 2bc) as well as the reconstruction of the bands themselves (Fig. 2a). Different excitation patterns from the valence band to the conduction band were observed, as a consequence of the photon energy of the laser. Along the considered k-path, the 2.0 eV laser pulse (P_B_) induced transitions at a few well-localized hot spots between bands near the ground state Fermi level ${E}_{{{\rm{F}}}}^{0}$ (Fig. 2c). Elsewhere, the change in occupancy is negligible or non-existent. We refer to the energy relative to the ground state Fermi level ${E}_{{{\rm{F}}}}^{0}$ as all occupations originate from the projection of the time-evolved states to the initial ground state electronic states. Conversely, the stronger 3.9 eV pulse (P_A_) caused excitation into slightly higher conduction bands due to the higher laser pulse energy, resulting in occupancy modifications spreading nearly across the entire studied k-path (Fig. 2b). In both cases, similar valence bands were depleted. However, for the 3.9 eV laser pulse (Fig. 2b), more transitions occurred due to the increased flatness of the higher conduction bands. Additionally, a larger amount of energy, expressed by a higher laser fluence, was delivered by the pulse P_A_. Nevertheless, the occupancy near the WNs changed only marginally (Fig. 2b, c).

Along with the changes in occupancy, a reconstruction of the band structure took place (Fig. 2a), where the time-dependent bands relate to the eigenvalue spectra of the Houston states (Equation (13))^27,28^. Generally, a non-uniform, momentum-dependent, shift of the electronic bands towards higher energy was observed. This effect is more pronounced with higher laser fluence as the TaAs system absorbs more energy. However, no strong bending of the bands was observed.

#### Dynamics of Weyl nodes

**Fig. 3:**
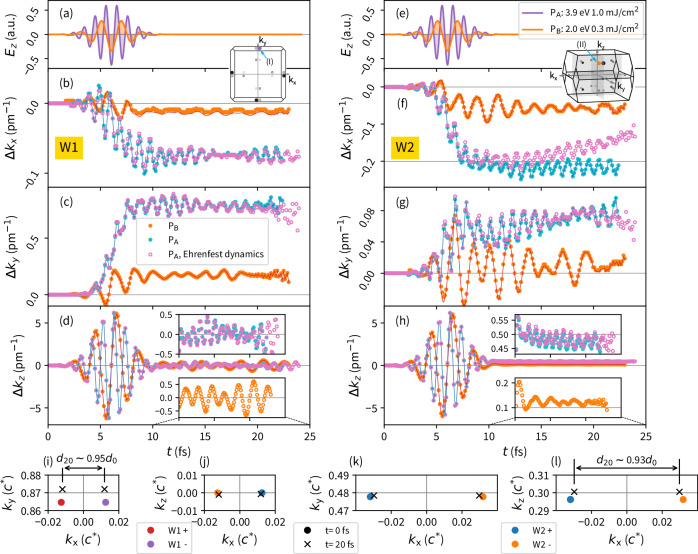
Weyl node dynamics in the *k*-space. TaAs Weyl-nodes' position time evolution in k-space. **a**, **e** Applied field. **b**–**d** W1 nodes. **f**–**h** W2 nodes. (points) WN positions determined from total phase difference *γ* and (lines) from related Weyl cone position in the band structure are depicted. (filled points and lines) No ion dynamics included. (empty points) Ehrenfest dynamics included. **i**–**l** Snapshot of the Weyl nodes positions. (bullet) Weyl nodes' positions at *t*=0 fs resp. (cross) positions at *t*=20 fs. *d*_0_, *d*_20_ denote WN separation at *t*=0 fs resp. *t*=20 fs. Two different pulse strengths (P_A_ and P_B_) with the field parallel to the *k*_*z*_ axis are assumed. Cartesian axes are considered. Weyl nodes whose dynamics were studied are denoted in the insets by arrows: W1 (I), W2 (II).

**Fig. 4:**
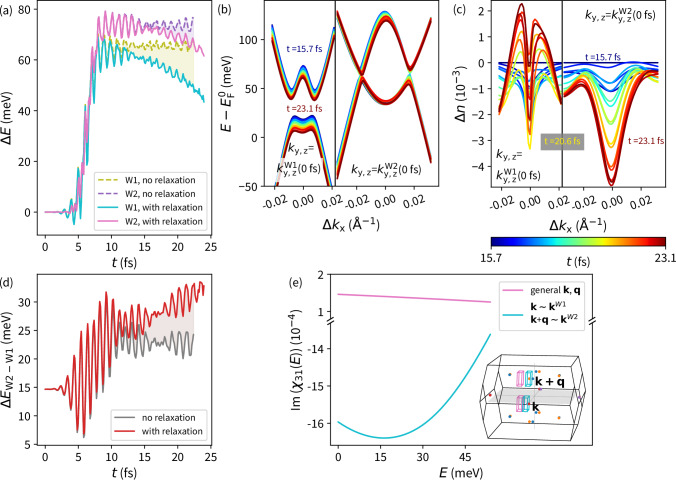
Modification of the Weyl nodes’ energy levels. **a** Laser pulse-induced change of the WNs' energy levels with respect to the application of the Ehrenfest dynamic. (solid line) relaxation included, (dashed line) without relaxation. **b** Band structure evolution near the W1 and W2 WNs. Ehrenfest dynamics included. **c** Time dependent change of the total occupation at two bands related to the Weyl cones depicted in the subfigure (**b**). The time stamp is denoted by different colors of the lines. **d** Laser pulse-induced change in the energy distance between the W1 and W2 Weyl nodes with respect to the applied relaxation. Laser pulse P_A_ is considered. **e** Imaginary part of the spin dependent response function *χ*_31_ component (Equation (24)). (pink curve) Off-resonant q-vector, k-points integrated out of the WNs pair as depicted in the Brillouin zone inset by pink boxes. No transition detected. (cyan curve) Resonant q-vector, and k-points integrated in the vicinity of the neighboring W1 and W2 Weyl nodes denoted by cyan boxes in the inset. A transition between W1 and W2 was detected.

Regardless of the field strength, similar qualitative dynamics of the WN positions was detected. The laser pulse-induced modifications in the band structure (Fig. 2a) led to shifts and oscillations of the WNs’ positions in k-space (Fig. 3) and changes in the W1 and W2 energy levels (Fig. 4a). The largest oscillations occurred during the pulse in the *k*_*z*_-direction, parallel to the laser pulse field (Fig. 3d, h). These significant oscillations can be attributed to the Stark shift. Comparing the induced shift to the applied effective electric field, the WN position displacement tends to follow the direction of the electric field *E*_z_ (Fig. 3a, e). Irrespective of the pulse strength (P_A_ vs. P_B_), the oscillations exhibit similar magnitudes due to almost identical vector field amplitudes *A*_z_. Consequently, the integral of the *E*_z_ field component over the half-period, driving the shift, is comparable. The WNs’ positions do not oscillate solely in the *k*_z_ direction; oscillations also occur in other directions (Fig. 3). For simplicity, Cartesian axes are considered instead of non-orthogonal reciprocal axes. We note that the oscillations persist after the laser pulse, maintaining a similar period. The non-vanishing oscillations result from the system’s response to the applied pulse and relaxation of the excited state. This is manifested in the total current (Fig. SI6), showing ongoing charge redistribution as the system attempts to reach equilibrium.

However, a more substantial detected feature is an induced displacement of the WN mean position (Figure 3i-l). This displacement propagates during the laser pulse, and a residual shift remains even after the pulse. The displacement is well-pronounced in the *k*_x_ and *k*_y_ directions. In contrast, for the *k*_z_ direction, the onset is overwhelmed by the initial significant oscillations during the laser pulse, and only a residual displacement is noticeable. We examined the field-induced displacement for both types of WNs. For the W1 WNs, which lie in the *k*_x_*k*_y_ plane, a significant residual displacement was observed for both in-plane components. We note that the displacements shown in Fig. 3 are related to the WNs depicted in the insets. Comparing the dynamics of several W1 nodes within the plane (Fig. SI4), the most dominant effect is shifting the WNs’ positions away from the BZ center (*Γ*-point). Meanwhile, the WN pair gets closer as their separation in k-space decreases. Due to the zero *k*_*z*_ component, restricted by symmetries, no residual shift occurs in this direction. Similarly, the W2 nodes are gathered by the laser pulse, where the relative change in the nodes’ separation corresponds to W1 nodes (Fig. 3i–l).

**Fig. 5:**
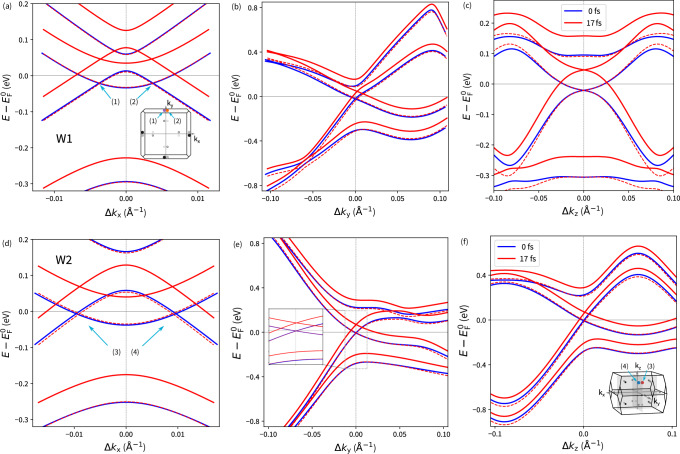
Weyl node *k*-position shift induced by a band reconstruction. **a**–**c** W1, **d**–**f** W2. Band structure in the vicinity of Weyl nodes at the (solid blue) *t*=0 fs and (solid red) *t*=17 fs are compared, where k-axes are centered at the initial ground state WN position. For clarity, (dashed line) time evolved band structures shifted by the energy difference at the WN with respect to the initial state are depicted. The P_A_-pulse is considered. Positions of the selected W1 and W2 WN pairs in the BZ are depicted in the insets.

The detailed origin of the WN position shift results from the time-dependent band structure reconstruction depicted in Fig. 5. Since it is challenging to handle the shifted Weyl cones, we compared the band structure evolution along the Cartesian axes near the WNs. For the W1 nodes, it is evident that the band structure is almost unchanged along the *k*_z_-direction near the WN, except for the energy shift caused by the laser pulse energy. The initial band separation and curvature are maintained for the selected time step. Lying in the *k*_x_*k*_y_ plane, the band structure respects the TaAs symmetry, preventing the WN position from shifting out of this plane. The WN shift in the *k*_x_ and *k*_y_ directions can be explained by a laser-induced separation of the valence and conduction bands towards the *Γ*-point, moving the WNs, as band crossings, out of the *Γ*-point in the *k*_y_ coordinate and simultaneously shrinking the WNs’ separation along the *k*_x_ direction (Fig. 5). For the W2 nodes, a similar model applies. Only near the W2 WNs, the band structure reconstruction along the *k*_y_ direction is negligible, with the dominant WN displacement occurring along the *k*_z_ component due to band energy modifications. The induced residual WN displacement in k-space occurs even at a much lower laser fluence. Although the related electron excitations are not highly significant (Fig. 2bc), the induced WNs’ displacements are large (Fig. 3). They follow previously described behavior, and the oscillations observed in the displacement correspond to the relevant laser pulse frequency.

For the stronger 3.9 eV pulse P_A_, the maximal magnitude of the residual WN displacement Δ_WN_ is reached by the W1 node Δ_W1_ ~ 0.8%*k*_c_, regarding the 2.0 eV pulse P_B_, Δ_W1_ ~ 0.25%*k*_c_ (Fig. 3b–d). Along with the modification of WNs’ k-space positions, the WNs’ energy levels are also altered by the laser pulse (Fig. 4a). Interestingly, the energy separation between the W1 and W2 WNs changes as well (Fig. 4d), with the laser pulse enhancing their separation by about 10 meV. This significant change in the WNs’ energy separation should be more apparent in experiments than slight modifications of the WNs’ positions.

Due to the complex spin texture related to the occurrence of WNs, we investigated signatures in the spin response function (Fig. 4e) (Equation (24)) that may originate from the presence of WNs. The significant modifications in the separation of the WNs (Figs. 3b–d, f–h and 4e), may be reflected in the response function, suggesting direct probing of the WN dynamic. For simplicity, the nearest W1 and W2 possessing opposite chirality were chosen in calculations (Fig. 4e). To identify the origin of the response features, we considered only a small segment of the BZ (Figure 4e-inset). Assuming the exact q-vector connecting the W1 and W2 WNs and k-points in their vicinity – resonant q-vector (Fig. 4e - inset cyan boxes), a transition (Fig. 4e - cyan curve) at the energy separation of the WNs (Fig. 4d) was observed. Comparison with response functions for a q-vector tilted from the resonant orientation and integrated across different k-space segments (Fig. 4e - pink curve and boxes) suggests a relation of the observed absorption feature (Fig. 4e - cyan curve) to a W1 and W2 transition. In the performed ground state calculations, we demonstrated that the transition follows the WNs’ energy separation when the resonant q-vector is considered. Thereby, for time-dependent WNs dynamics, it can be assumed that the energy of the transition should follow the WNs energy separation in time, as well as the resonant q-vector should correspond to changing WNs positions.

#### Relaxation effects

Thus far, we have considered fixed lattice sites during the time evolution. Consequently, after the laser pulse, the electronic subsystem appears to reach a quasi-equilibrium state, with the resulting residual WN displacement (Fig. 3) and WNs’ energy levels (Fig. 4a) remaining almost unchanged with increasing time delay due to the inability to dissipated energy to other degrees of freedom. Therefore, it is crucial to allow the ions to move from their ground state equilibrium positions. For this purpose, we incorporated Ehrenfest dynamics (Equation (22)), allowing a coupling between the excited electronic systems and the lattice. This enables the electronic system to dissipate the acquired energy to lattice vibrations and progress towards the initial state. Including ion motion, the WN dynamics, whether in energy or k-space, was relatively unaffected for times less than *t* = 10 fs, as it is driven by the applied laser pulse (Figs. 3, 4a). Subsequently, significant relaxation processes in the WNs’ k-space and energy positions occurred as lattice vibrations are excited. Their onset is also apparent from the laser pulse-induced current (Figure SI6), where the evolution starts to deviate from calculations neglecting ion motion, indicating changes in electron density. Relaxation effects are pronounced particularly in changes in the WNs’ energy levels (Fig. 4a) and their resulting separation (Fig. 4d). Unlike calculations without Ehrenfest dynamics, the WN energy levels tend to return to their original positions after the laser pulse (Figure 4a). Moreover, faster relaxation of W1 WNs due to a stronger dissipation of energy from the associated electronic bands, in fact results in the growth of the WNs’ energy separation (Fig. 4d). The difference between the energy relaxation of W1 and W2 WNs is likely connected to distinct changes in band occupations. Assuming a k-path in the vicinity of the WNs (Fig. 4b), the occupancy (Fig. 4c) summed over the bands related to WNs (Fig. 4b) revealed ongoing depleting of bands forming W2. However, regarding W1 WNs, a growth of the occupation is initiated around the times when the WNs’ energy separation begins to increase (Fig. 4d).

Besides energy relaxation, shifts in the WNs’ k-space positions also occur, particularly for the W2 nodes and their *k*_*x*_ component (Fig. 3f). An apparent reduction in the residual displacement is observed beyond 15 fs. Further, the onset of relaxation is visible in the *k*_*y*_ direction at later times (Fig. 3g). Although less evident, relaxation effects also influence W1 nodes’ positions (Fig. 3c). The shifting of the WN positions due to relaxation is related to previously discussed modifications in the band structure reconstruction (Fig. 5).

**Fig. 6:**
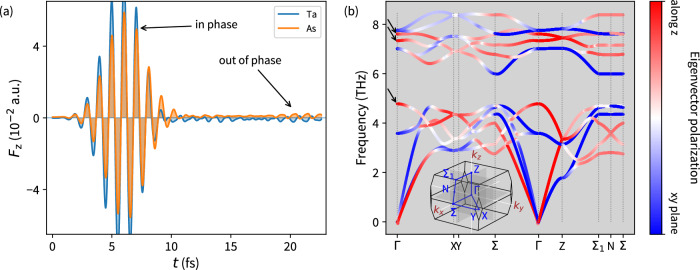
Lattice dynamics. **a** Time-dependent forces acting on the Ta and As sites. Laser pulse P_A_ is considered. **b** Eigenvector polarization in the TaAs phonon band structure along high symmetry points. Arrows denote optical modes oscillating parallel to the applied laser field (at *Γ* point).

Evaluated forces acting on the atoms (Fig. 6a) indicate the relation of relaxation effects to the dissipation of energy from the electronic degrees of freedom to phonons. During the laser pulse, the atoms are pushed to follow the applied linearly polarized field and oscillate in phase. Later, through a complex evolution, nearly constant forces occur around 15 fs as a quasi-equilibrium state is reached. Interestingly, beyond 19 fs, out-of-phase oscillations arise at a similar time delay where the onset of relaxation effects in the current or the WN positions is observed. This can be ascribed to the occurrence of optical phonons, the opposite direction of Ta and As forces, through electron-phonon coupling. Similar laser-induced non-equilibrium processes, including the interplay of the electron and phonon baths, have been studied by ultrafast electron diffraction and numerical simulations^29,30^. With forces predominantly pointing along the *z*-direction, we tried to identify the phonon modes likely taking part in this effect. Considering the phonon eigenvector polarization at the *Γ*-point, we denote modes with *z*-polarization by arrows in Fig. 6b. We note that the force oscillations have much higher frequency than the denoted phonon modes with *z*-polarization. Nevertheless, even an off-resonant interaction enables dissipation of energy from the electronic to the ionic system.

Laser-induced reduction of the WN k-space separation has been experimentally reported for the more complicated WTe_2_ system^4^ on much longer time scales, where it is explained by induced shear modes modifying the lattice. Our calculations provide a similar effect, describing the laser-induced band structure relaxation, giving rise to the shifting of the WN nodes as well as relaxation due to lattice dynamics. In the studied cases, no annihilation of WNs due to the laser pulse was observed within the time-scale of the simulation. The presence of WNs was confirmed using Equation (18) (Fig. 3) within the accessible time range. A promising approach to indirectly investigate Weyl-point dynamics on ultrafast timescales involves second harmonic generation (SHG). Sirica et al. demonstrated a pronounced spectral sensitivity of TaAs to this technique^31^. An interesting direction for future research would be to compute the transient SHG response as a function of incident light polarization and directly compare these results with experimental spectra. Such a comparison could reveal the laser-induced band shifts and provide insight into the fluence and photon energy thresholds required to drive a Lifshitz transition in TaAs specifically through symmetry-breaking processes intrinsic to the electronic structure^19^.

In conclusion, we show that laser-induced band-structure renormalizations may lead to significant changes in the position of topologically protected Weyl nodes, even without significant structural changes. Focusing on the TaAs Weyl semimetal, motion in momentum space for both types of WN pairs, along with long-lasting modifications of the WNs’ energy levels and their separation were observed. Notably, the induced changes persist even after the pulse duration. The evidence of laser pulse-induced WNs displacement aligns with experimental results showing decreasing separation of WNs in other Weyl materials. We demonstrated the influence of ion dynamics on the relaxation of WN dynamics through energy dissipation to the lattice, essential for following the relaxation pathways of the system towards the ground state location of the WNs. Our demonstrated ability to track WN motion in real-time using time-dependent density functional theory facilitates the development of pulse shaping techniques for optimal control pre-programming, paving the way to energy-efficient computing.

## Methods

### Crystal structure

The non-trivial band structure topology of TaAs, which crystallizes in the tetragonal I4_1_md space group lacking the xy-mirror plane, results in the emergence of robust Weyl cones with linear dispersion, hosting chiral massless Weyl quasiparticle states. The broken crystal inversion symmetry, characteristic of nonmagnetic Weyl materials, plays a crucial role in its physical properties. In the performed calculations, the experimental crystal structure^32,33^ was considered. Regarding a tetragonal cell, it reads *a* = 3.4348 Å, *c* = 1.1641 Å (Figure SI1).

### Ab-initio calculations

The calculations provided in this work were performed within the *Elk* code^24^, the all-electron full-potential linearized-augmented-plane-wave (LAPW)^34^ package. It represents a robust and powerful open-source tool, which allows us to treat the ground state density functional theory (DFT) calculations, as well as more advanced features *i.e*. real-time time-dependent density functional theory (TDDFT) system evolution^35^ and linear response calculations^36^. To study the time-dependent band structure and Weyl node (WN) dynamics, we used an approach described below in this section. Ground state DFT calculations were performed on the 12 × 12x12 k-mesh, while the exchange correlation potential in the generalized gradient approximation (GGA) of Perdew-Burke-Ernzerhof (PBE)^37^ type was included. Regarding the TDDFT evolution calculations, the same parameters were used as for the ground state, including the adiabatic GGA (AGGA) xc-potential approach.

### DFT formalism

The ground state in the framework of the *Elk* code is determined by the common Kohn-Sham (KS) equation^38,39^

1 $$\left(-{\nabla }^{{{\rm{2}}}}+{v}_{{{\rm{ext}}}}({{\bf{r}}})+\int\,\frac{n({{{\bf{r}}}}^{{\prime} })}{| {{\bf{r}}}-{{{\bf{r}}}}^{{\prime} }| }{{\rm{d}}}{{{\bf{r}}}}^{{\prime} }+{v}_{{{\rm{xc}}}}({{\bf{r}}})\right){\varphi }_{{{\rm{i}}}}({{\bf{r}}})={\varepsilon }_{{{\rm{i}}}}{\varphi }_{{{\rm{i}}}}({{\bf{r}}})\,,$$

where *v*_ext_ is an external potential, *v*_xc_ exchange correlation potential, *n*(**r**) represents the single particle electron density and *ε*_i_ stands for the eigenenergy of the KS state *φ*_i_. We note that we are using the Hartree atomic units in expressions. In the *Elk*, the KS equations (Equation (1)) are solved in the following two variation step scheme. First, only scalar potentials are considered:

2 $${\hat{H}}^{{{\rm{I}}}}={\hat{T}}_{{{\rm{S}}}}+{\hat{V}}_{{{\rm{ext}}}}+{\hat{V}}_{{{\rm{C}}}}+{\hat{V}}_{{{\rm{XC}}}}$$

3 $${\hat{H}}^{{{\rm{I}}}}{\phi }_{{{\rm{i}}}}^{{{\rm{I}}}}={\epsilon }_{{{\rm{i}}}}^{{{\rm{I}}}}{\phi }_{{{\rm{i}}}}^{{{\rm{I}}}}\,,$$

${\hat{T}}_{{{\rm{S}}}}$ stands for the kinetic term, ${\hat{V}}_{{{\rm{ext}}}}$ is an external potential, ${\hat{V}}_{{{\rm{C}}}}$ denotes the Coulomb potential and ${\hat{V}}_{{{\rm{XC}}}}$ represents the xc-potential. To cover relativistic effects and the emergence of Weyl quasipartiles, considering spin-orbit coupling (SOC) is required^1,2^. It is included by means of the scalar relativistic approach together with the external and xc-magnetic fields (**B**_ext_ resp. **B**_xc_), and an applied vector potential **A** in the second variational step:

4 $$\begin{array}{l}{H}_{{{\rm{ij}}}}={\epsilon }_{{{\rm{i}}}}^{{{\rm{I}}}}{\delta }_{{{\rm{ij}}}}+\\ +\langle {\phi }_{{{\rm{i}}}}^{{{\rm{I}}}}| \hat{{{\boldsymbol{\sigma }}}}\cdot \left({\hat{{{\bf{B}}}}}_{{{\rm{ext}}}}+{\hat{{{\bf{B}}}}}_{{{\rm{xc}}}}\right)+\hat{{{\boldsymbol{\sigma }}}}\cdot \hat{{{\bf{L}}}}+{{\bf{A}}}\cdot \nabla | {\phi }_{{{\rm{j}}}}^{{{\rm{I}}}}\rangle \,,\end{array}$$

5 $$\hat{H}\left\vert {\phi }_{{{\rm{i}}}}^{{{\rm{II}}}}\right\rangle ={\epsilon }_{{{\rm{i}}}}^{{{\rm{II}}}}\left\vert {\phi }_{{{\rm{i}}}}^{{{\rm{II}}}}\right\rangle ={\epsilon }_{{{\rm{i}}}}^{{{\rm{II}}}}\mathop{\sum}\limits_{{{\rm{j}}}}{c}_{{{\rm{j}}}}^{{{\rm{II}}}}\left\vert {\phi }_{{{\rm{j}}}}^{{{\rm{I}}}}\right\rangle \,.$$

Since, generally, a non-collinear magnetism is considered, the second variational eigenvectors $| {\phi }^{{{\rm{II}}}}\left.\right\rangle$ are spinors, ${\epsilon }_{{{\rm{i}}}}^{{{\rm{II}}}}$ denotes related eigenenergies and $\hat{{{\boldsymbol{\sigma }}}}$ stands for Pauli matrices. For simplicity, the second variational eigenvectors $| {\phi }^{{{\rm{II}}}}\left.\right\rangle$, diagonalizing total Hamiltonian *H* (Equation (5)), are represented in the first variational basis $| {\phi }_{{{\rm{j}}}}^{{{\rm{I}}}}\left.\right\rangle$ (Equation (3)) by coefficients *c*^II^ (Equation (5)).

### TDDFT formalism

The time-evolution of the ground state wave functions is considered as a simple direct propagation without self-consistent treatment in time^40^. An evolution of a KS state $| \varphi (t)\left.\right\rangle$ in the time difference d*t* reads

6 $$\left\vert \varphi (t+{{\rm{d}}}t)\right\rangle =\hat{U}(t)\left\vert \varphi (t)\right\rangle \,,$$

where $\hat{U}(t)$ is the evolutionary operator

7 $$\hat{U}(t)=\exp \left[-i\hat{H}(t){{\rm{d}}}t\right]$$

related to the instantaneous Hamiltonian $\hat{H}(t)$ at the time *t*.

Assuming the *velocity gauge*^40–42^, we neglect spatial dependencies of the vector potential **A** and impose the Coulomb gauge condition ∇ ⋅ **A** = 0. Then, in the *dipole approximation* and the second variational basis $| {\phi }_{{{\rm{j}}}}^{{{\rm{II}}}}\left.\right\rangle$ (Equation (5)), the Hamiltonian matrix elements read

8 $$\quad {H}_{{{\rm{ij}}}}(t)={V}_{{{\rm{S}}}({{\rm{ij}}})}(t)+{T}_{{{\rm{S}}}({{\rm{ij}}})}(0)-{{\bf{A}}}(t)\cdot {{{\bf{P}}}}_{{{\rm{ij}}}}(0)\,,$$

where *V*_S_(*t*) denotes Kohn-Sham potential related to eigenstates $| {\phi }_{{{\rm{i}}}}^{{{\rm{II}}}}(t)\left.\right\rangle$, *T*_S(ij)_(0) is the initial kinetic part (Equation (9)) and the final term stands for the interaction with the external vector potential **A**(*t*) using the momentum matrix **P**_*ij*_.

9 $${T}_{{{\rm{S}}}({{\rm{ij}}})}={\varepsilon }_{{{\rm{i}}}}^{{{\rm{II}}}}{\delta }_{{{\rm{ij}}}}-\langle {\phi }_{{{\rm{i}}}}^{{{\rm{II}}}}| {V}_{{{\rm{S}}}}| {\phi }_{{{\rm{j}}}}^{{{\rm{II}}}}\rangle ,$$

10 $${{{\bf{P}}}}_{ij}=\int\,{d}^{3}r\,{\phi }_{i{{\bf{k}}}}^{{{\rm{II}}}* }({{\bf{r}}})\left(-i\nabla +\frac{1}{4}\left[\overrightarrow{\sigma }\times \nabla {V}_{{{\rm{S}}}}({{\bf{r}}})\right]\right){\phi }_{j{{\bf{k}}}}^{{{\rm{II}}}}({{\bf{r}}})\,,$$

11 $$\begin{array}{l}{{\bf{A}}}(t)=-\int_{0}^{t}{{\bf{E}}}(\tau ){{\rm{d}}}\tau \\ \hslash ,c,e=1\,{,}\end{array}$$

In this work, we considered a time evolution induced by a linearly polarized laser pulse. It is described by a vector potential **A**(*t*) constructed from a sinusoidal wave modulated with a Gaussian envelope function

12 $${{\bf{A}}}(t)={{{\bf{A}}}}_{0}\frac{{e}^{-{(t-{t}_{{{\rm{p}}}})}^{2}/2{\sigma }^{2}}}{\sigma \sqrt{2\pi }}\sin \left[\omega (t-{t}_{{{\rm{p}}}})+\phi \right]\,$$

parameterized by the vector amplitude **A**_0_, peak time *t*_p_, full-width at half-maximum $d=2\sqrt{2\ln 2}\sigma$, frequency *ω* and phase shift *ϕ*.

Diagonalizing the time-dependent Hamiltonian (Equation (8)), one obtains third variational vectors $| {\phi }_{{{\rm{i}}}}^{{{\rm{III}}}}(t)\left.\right\rangle$, so-called *Houston states*^27,28^, with eigen-energies ${\epsilon }_{{{\rm{i}}}}^{{{\rm{III}}}}(t)$

13 $${H}_{{{\rm{ii}}}}(t)\,\left\vert {\phi }_{{{\rm{i}}}}^{{{\rm{III}}}}(t)\right\rangle ={\epsilon }_{{{\rm{i}}}}^{{{\rm{III}}}}(t)\,\left\vert {\phi }_{{{\rm{i}}}}^{{{\rm{III}}}}(t)\right\rangle \,.$$

Then, the evolved states are given by a simple formula

14 $$\begin{array}{ll}\left\vert {\phi }_{{{\rm{i}}}}^{II}(t+{{\rm{d}}}t)\right\rangle =\\=\mathop{\sum}\limits_{i}{{{\rm{e}}}}^{-i{\varepsilon }_{{{\rm{j}}}}^{{{\rm{III}}}}(t){{\rm{d}}}t}\langle {\phi }_{{{\rm{i}}}}^{II}(t)| {\phi }_{{{\rm{j}}}}^{{{\rm{III}}}}(t)\rangle \,\left\vert {\phi }_{{{\rm{i}}}}^{II}(t)\right\rangle \,.\end{array}$$

The most straightforward way to track the band structure evolution might be following an evolution of ground state wave functions (Equation (5)) and evaluation of the expectation values of the instantaneous Hamiltonian $\hat{H}(t)$ (Equation (8)) related the time evolved states $| {\phi }_{{{\rm{i}}}}^{{{\rm{II}}}}(t)\left.\right\rangle$ as follows

15 $${\varepsilon }_{{{\rm{i}}}}^{{{\rm{II}}}}(t)=\langle {\phi }_{{{\rm{i}}}}^{{{\rm{II}}}}(t)| \hat{H}(t)| {\phi }_{{{\rm{i}}}}^{{{\rm{II}}}}(t)\rangle \,.$$

Let’s call this approach a ground-stated evolved band structure. Nevertheless, the given approach fails in the presence of inter-band transitions. In such a case, there arises an interchange of contributions to the expansion coefficients

16 $$\left\vert {\phi }_{{{\rm{i}}}}^{{{\rm{II}}}}\right\rangle (t)=\mathop{\sum}\limits_{j}{c}_{{{\rm{j}}}}^{{{\rm{III}}}}(t)\left\vert {\phi }_{{{\rm{j}}}}^{{{\rm{III}}}}\right\rangle (t)$$

between the coupled bands, leading to an energy shift of particular bands. Mixing the expansion coefficients, the related band energies are corrupted locally, which gives rise to an artificial twisting of the band structure.

Regarding failures of the progressively evolved band structure from ground state, it is more appropriate to determine the band structure based on the time-dependent eigenvalue spectrum (Equation (13)) of the instantaneous Hamiltonian *H*_ij_(*t*) (Equation (8)) defining system’s instantaneous states – the Houston states $\left\vert {\phi }_{{{\rm{i}}}}^{{{\rm{III}}}}(t)\right\rangle$. Their occupancies and character are obviously determined by projections to the second variational basis $\langle {\phi }_{{{\rm{i}}}}^{{{\rm{II}}}}(t)| {\phi }_{{{\rm{i}}}}^{{{\rm{III}}}}(t)\rangle$, assuming the initial occupancy and character of the initial states $| {\phi }_{{{\rm{i}}}}^{{{\rm{II}}}}(0)\left.\right\rangle$ (Equation (14)). To study an evolution of the occupation numbers or band character along a selected k-path, an auxiliary k-set representing an arbitrary k-path must be included, as the evolution at particular k-points must be tracked from the initial step (Equation (6)) (Figure 2b, c). Possibly, an auxiliary k-mesh can be avoided if only a band structure spectrum without occupation numbers is desired. Storing the instantaneous charge density *n*(**r**, *t*), magnetic spin density *m*(**r**, *t*) and Kohn-Shame potential *V*_S_(*t*), the related instantaneous Hamiltonian $\hat{H}(t)$ (Equation (8)) for the applied vector field **A**(*t*). can be restored and used to determine eigenvalues ${\varepsilon }_{{{\rm{i}}}}^{{{\rm{III}}}}(t)$ along an arbitrary k-path.

It is worth mentioning that the densities *n*(**r**, *t*) and Kohn-Sham potential *V*_S_(*t*) were obtained for transient occupations at the time *t*. Therefore, for the applied vector field **A**(*t*), they directly determine the instantaneous states (Equation (13)) at the time *t*. All the potential and densities are related to an appropriate occupation arising from the TDDFT calculations. In our calculations, we applied a linearly polarized pulse along the *k*_*z*_ axis. It is the simplest choice as the field is parallel to the high-symmetry crystallographic z-axis and does not break the perpendicular plane symmetry. Therefore, related time evolution calculations are the most feasible ones from the point of view of computational demands. Originally, we had performed the TD-DFT calculations using a time step *δt* = 0.10 a.u. (~ 2.4 as) and laser pulse width FWHM ~ 7.3 fs. Nevertheless, the maximum time-delay is limited by available computational resources and numerical stability. Even for the not large studied TaAs system, the TD-FT evolution suffered from numerical instability after a few fs. It is manifested by sudden scattering in the WN position evolution in the Figure SI2. This feature was sensitive to the time step length, and it could be removed by a shorter time step. Therefore, we squeezed the laser pulse width (FWHM ~ 3.6 fs) and considered a shorter time (*δt* = 0.05 a.u.), which provided us a longer time window to observe WN dynamics after the laser pulse. Two different strengths of the linearly polarized laser pulses were used to scale the observed effects. The stronger one with the laser fluence of 1.0 mJ/cm^2^ with the energy 3.9 eV (peak 7.4 ⋅ 10^11^ W/cm^2^) – called P_A_ and weaker possessing the fluence of 0.3 mJ/cm^2^ and energy 2.0 eV (peak 2.2 ⋅ 10^11^ W/cm^2^) – called P_B_ (Fig. 3).

### Weyl nodes’ dynamics

Weyl nodes (WN) represent monopoles and antimonopoles of the Berry curvature^1–3,15^. Their presence is characterized by a non-vanishing topological invariant, so-called Chern number *C*^1^. At Weyl nodes, it acquires non-zero values depending on the vortex character. The Chern number is defined by means of the total Berry flux ${{\mathcal{F}}}({{\bf{k}}})$ over a closed surface in the k-space, which results in a gauge invariant variable^1,5,43,44^

17 $$C=\frac{1}{2\pi }\oint {{\rm{d}}}{{\bf{k}}}\,{{\mathcal{F}}}({{\bf{k}}}).$$

Assuming a 2D k-space, the Chern number *C* can be also attributed to the phase *γ*^43,45,46^ picked up during the *parallel transport*^10,47^ along a close loop as follows

18 $$C=\frac{1}{2\pi }\gamma .$$

Considering fine discrete k-mesh and single band, the total phase difference *γ* along a close loop represented by a set of k-points {**k**_1_, **k**_2_, …, **k**_M_, **k**_1_} reads^43,45,46,48^

19 $$\gamma ={{\rm{Im}}}\log \left[\mathop{\prod}\limits_{i=1}^{M}{U}_{i+1,1}\right];M+1\equiv 1\,,$$

20 $${U}_{i+1,i}=\frac{\langle \varphi ({{{\bf{k}}}}_{i+1})| {{{\rm{e}}}}^{i{{\bf{q}}}{{\bf{r}}}}| \varphi ({{{\bf{k}}}}_{i})\rangle }{| \langle \varphi ({{{\bf{k}}}}_{i+1})| {{{\rm{e}}}}^{i{{\bf{q}}}{{\bf{r}}}}| \varphi ({{{\bf{k}}}}_{i})\rangle | };\,{{{\bf{k}}}}_{i+1}={{{\bf{k}}}}_{i}+{{\bf{q}}}$$

where *U* is a link variable defined by an overlap of the wave function *φ* at the ends of the segment *i*. Importantly, unlike the phase difference along particular segments, the total phase difference *γ* is a gauge-invariant quantity and represents an observable. Regarding a larger system with *N* occupied band, the link variable becomes a determinant of *N* × *N* matrix with elements defined as follows^43,46,48^

21 $${U}_{i,i+1}=\det \frac{\langle \varphi ({{\bf{k}}}+{{\bf{q}}})| {{{\rm{e}}}}^{i{{\bf{q}}}{{\bf{r}}}}| \varphi ({{\bf{k}}})\rangle }{| \langle \varphi ({{\bf{k}}}+{{\bf{q}}})| {{{\rm{e}}}}^{i{{\bf{q}}}{{\bf{r}}}}| \varphi ({{\bf{k}}})\rangle | }\,,$$

where *m*, *n* denote band indices. Considering a bulk system, the mentioned approach can be applied to determine the Berry flux flowing through a closed loop. Then, Weyl nodes’ presence and their positions can be traced by searching for discontinuities in the Berry flux ${{\mathcal{F}}}({{\bf{k}}})$ along different directions in the k-space^48,49^.

In general, in this paper, we integrated along squared loop with an edge size down to *l* ~ 4 ⋅ 10^−2^ Å^−1^ and k-resolution down to Δ*k* ~ 10^−4^ Å^−1^. We considered phase integration over the 84 lowest-lying bands representing the occupied states in the ground state. We note that despite the laser-induced excitations over the ground state Fermi level ${E}_{{{\rm{F}}}}^{0}$ (Fig. 2), the same number of bands were used in the evaluation of the WNs at *t* > 0 as those bands are still predominantly occupied.

To efficiently determine the positions of the WNs, we initially divided the BZ into several slabs for rough localization (Equation (18)). Subsequently, by reducing the size (Equation (19)) of the Wilson loop^25^, we traced the WNs more accurately. It is noteworthy that the determined WN positions are significantly influenced by the loop size, which manifests as shifts in the position of the discontinuity in the integrated phase along a studied direction in k-space (Figure SI3). However, convergence with respect to k-spacing can be achieved. For overly large loops, a not well-separated WN pair might be hidden as the difference is integrated out.

To verify the WNs’ dynamics obtained through discontinuities in Berry flux, we searched for the Dirac cones in the vicinity of the resulting positions. Considering band structures along Cartesian axes (Fig. 5), we iterated up to almost touching bands (Δ*E* < 10^−6^ eV)(Fig. 1). WNs k-space positions from the phase difference *γ* and band crossing are compared in Fig. 3. The localization of Dirac cone positions in the band structure allowed us to follow the WN dynamics in the energy space as well (Fig. 4a).

### Lattice dynamics

Initially, the mentioned experimental crystal structure^32,33^ was considered, neglecting the laser induced ions motion through the time evolution. Later, to examine the effect of the lattice relaxation, Ehrenfest dynamics was included in the TDDFT calculations. A simple approach, included in the *Elk*, was applied to cover modifications of the nuclear Coulomb potential caused by atomic displacement. It includes an extra contribution to the Coulomb potential based on the gradient of the initial nuclear potential and displacement arising from previously evaluated time-dependent inter-atomic forces^50,51^.

22 $${V}_{{{\rm{S}}}}({{\bf{r}}},t)\to {V}_{{{\rm{S}}}}({{\bf{r}}},t)-\mathop{\sum}\limits_{z,\alpha }\frac{\partial {V}_{{{\rm{N}}}}({{\bf{r}}})}{\partial {u}_{z,\alpha }}\delta {u}_{z,\alpha }$$

### Phonon spectra

To get insight into the phonon modes which might be responsible for the relaxation effects, we calculated the phonon spectra (Fig. 6b) using the finite displacement method employing *phonopy*^52,53^ – a python-based package. Electron structure calculations were performed within the *Elk* code using the same settings as for the TD-DFT calculations and considering 2 × 2 × 2 supercells based on the relaxed structure. Depicting the eigenvector polarization, we indicated modes which might interact with the laser pulse.

### Spin dependent response function

The occurrence of the WNs is related to the presence of chiral states and complex spin structure. The studied TaAs Weyl semimetal possesses two sets of the WNs, W1 resp. W2, lying at distinct energy levels. The energy separation between the W2 and W1 WNs is roughly 15 meV in the ground state (Fig. 4d), and it is enlarged by the applied laser pulse. Then, inter-band transitions between those WNs at distinct energies before and after the laser pulse might be observed. To identify them, we evaluated the spin-dependent KS response function defined in the real space and frequency domain as follows^24,54,55^

23 $$\begin{array}{ll}{\chi }_{\alpha \beta ,{\alpha }^{{\prime} }{\beta }^{{\prime} }}({{\bf{r}}},{{{\bf{r}}}}^{{\prime} },\omega )\equiv \frac{\partial {\rho }_{\alpha \beta }({{\bf{r}}},\omega )}{\partial {\nu }_{{\alpha }^{{\prime} }{\beta }^{{\prime} }}({{{\bf{r}}}}^{{\prime} },\omega )}=\\\,=\frac{1}{{N}_{k}}\mathop{\sum}\limits_{i{{\bf{k}}},j{{{\bf{k}}}}^{{\prime} }}\left({f}_{i{{\bf{k}}}}-{f}_{j{{{\bf{k}}}}^{{\prime} }}\right)\frac{\langle i{{\bf{k}}}| {\hat{\rho }}_{\beta \alpha }({{\bf{r}}})| j{{{\bf{k}}}}^{{\prime} }\rangle \langle j{{{\bf{k}}}}^{{\prime} }| {\hat{\rho }}_{{\alpha }^{{\prime} }{\beta }^{{\prime} }}({{{\bf{r}}}}^{{\prime} })| i{{\bf{k}}}\rangle }{\omega +({\varepsilon }_{i{{\bf{k}}}}-{\varepsilon }_{j{{{\bf{k}}}}^{{\prime} }})+i\eta },\end{array}$$

where *α*, *β* stands for spinor components, *ρ* is the spin-density, *ν* denotes the Kohn-Sham potential *N*_*k*_ is number of k-points and *f*_*i***k**_ is the occupancy of the state *i* at the k-point *k*. $| i{{\bf{k}}}\left.\right\rangle$ denotes KS states with related eigen-energies *ε*_*i***k**_, and *η* is a small real positive number. Applying the Fourier transformation, the response function in the reciprocal space reads

24 $$\begin{array}{ll}{\chi }_{\alpha \beta ,{\alpha }^{{\prime} }{\beta }^{{\prime} }}({{\bf{G}}},{{{\bf{G}}}}^{{\prime} },{{\bf{q}}},\omega )=\\ =\frac{1}{\Omega {N}_{k}}\mathop{\sum}\limits_{i{{\bf{k}}},j{{\bf{k}}}+{{\bf{q}}}}\left({f}_{i{{\bf{k}}}}-{f}_{j{{\bf{k}}}+{{\bf{q}}}}\right)\frac{{\left[{Z}_{i{{\bf{k}}},j{{\bf{k}}}+{{\bf{q}}}}^{\alpha \beta }({{\bf{G}}})\right]}^{* }\,{Z}_{i{{\bf{k}}},j{{\bf{k}}}+{{\bf{q}}}}^{{\alpha }^{{\prime} }{\beta }^{{\prime} }}({{{\bf{G}}}}^{{\prime} })}{\omega +({\varepsilon }_{i{{\bf{k}}}}-{\varepsilon }_{j{{\bf{k}}}+{{\bf{q}}}})+i\eta };\end{array}$$

25 $${Z}_{i{{\bf{k}}},j{{\bf{k}}}+{{\bf{q}}}}^{\alpha \beta }({{\bf{G}}})=\int\,{{{\rm{d}}}}^{3}r\,{{{\rm{e}}}}^{i({{\bf{G}}}+{{\bf{q}}})\cdot {{\bf{r}}}}{\varphi }_{j{{\bf{k}}}+{{\bf{q}}},\alpha }^{* }({{\bf{r}}}){\varphi }_{i{{\bf{k}}},\beta }({{\bf{r}}}),$$

where **G** is the reciprocal lattice vector and Ω is the reciprocal volume. In Fig. 4e, the imaginary parts of the *χ*_31_ response component denote the response of the *m*_*z*_ component of magnetization to the *m*_*x*_ magnetization. Unlike other calculations, the LDA xc-potential was used as implemented in the *Elk* code. To evaluate the response function, the sum over **k**-vectors (Equation (24)) considers 12 × 12 × 12 k-mesh around a WN in a k-box limited by following vectors in Cartesian coordinates: ${k}_{{{\rm{1}}}}=(0.5,0,0){r}_{{{\rm{B}}}}^{-1}$, ${k}_{{{\rm{2}}}}=(0,0.135,0){r}_{{{\rm{B}}}}^{-1}$, ${k}_{{{\rm{3}}}}=(0,0.038,0.1){r}_{{{\rm{B}}}}^{-1}$.

To determine whether the response feature (Fig. 4e) originates from the WNs itself, we considered in our calculations only a small segment of the k-space containing the WNs (Fig. 4e – inset). We calculated the response for different parts of the k-space and different q-vector orientations. Choosing the q-vector pointing in between the WNs and k-points in their vicinity, a resonance in the imaginary part at the energy separation of the WNs was observed, indicating a transition between those WNs.

## Supplementary information

Supplementary

## Data Availability

The datasets and basic input files are available from the Zenodo repository 10.5281/zenodo.13928031.

## Code availability

Ab-initio calculations were performed within the open-source *Elk* code package^24^. Phonon spectra were evaluated by the *phonopy* package^52,53^. *Elk* code extensions may be made available to qualified researchers on reasonable request from the corresponding author.
