# Supplementary material for: Photo-induced manipulation and relaxation dynamics of Weyl-semimetals
Source: NPJ Comput Mater. 2025 Jul 7;11(1):219. doi: 10.1038/s41524-025-01708-0 (PMC12234347; doi:10.1038/s41524-025-01708-0)
Supplement: Supplementary file 1 — Supplementary [file 41524_2025_1708_MOESM1_ESM.pdf]

Supplementary Information
Photo-induced Manipulation and Relaxation Dynamics of Weyl-semimetals

Jakub Šebesta[1,2] and Oscar Grånäs[1*]

[1]Materials Theory, Department of Physics and Astronomy, Uppsala University, Box 516, Uppsala, 751 20 , Sweden.
[2]IT4Innovations, VSB – Technical University of Ostrava, 17. listopadu 2172/15, Ostrava-Poruba, 708 00, Czech Republic.

*Corresponding author(s). E-mail(s): oscar.granas@physics.uu.se;
Contributing authors: jakub.sebesta@vsb.cz;

1 TaAs srystal structure

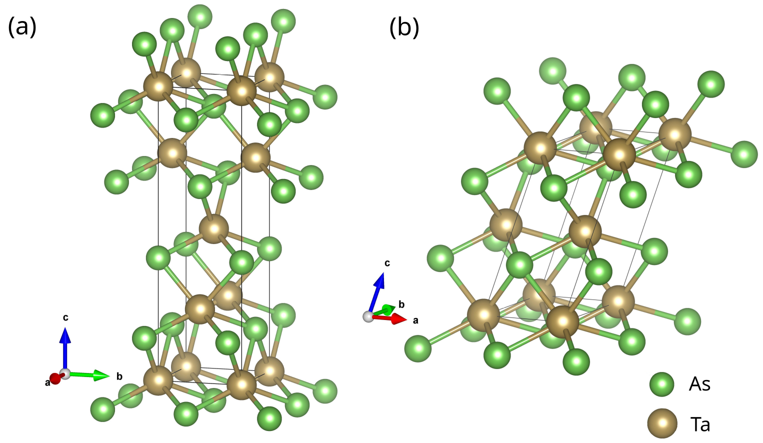

Fig. SI1 TaAs crystal structure. (a) tetragonal cell, (b) primitive cell used in *Elk* calculations. [1]

2 Numerical stability with respect to the time step

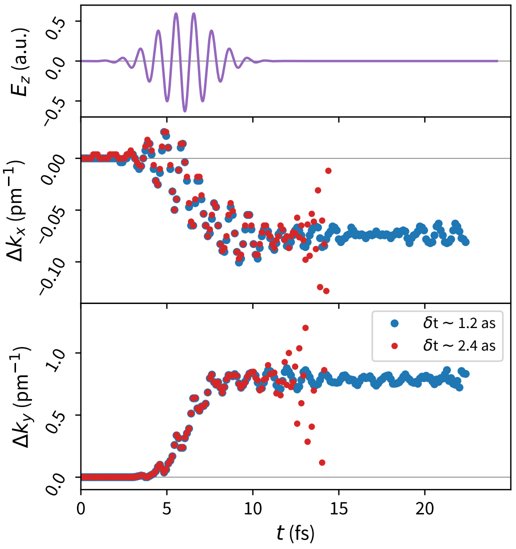

Fig. SI2 Influence of the time step length. Extracted Weyl node (WN) W1 position for different TDDFT time step lengths δt. Laser pulse P_A is considered.

3 Total phase difference convergence

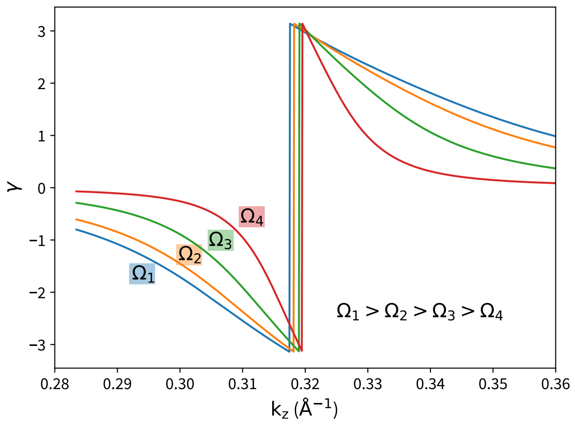

Fig. SI3 Wilson loop size effect. The influence of the Wilson loop size on the integrated total phase difference γ as a function of the k_z position. Ω_i denotes the area of the loop.

4 Dynamics in the k-space of the selected Weyl nodes across the Brillouin zone

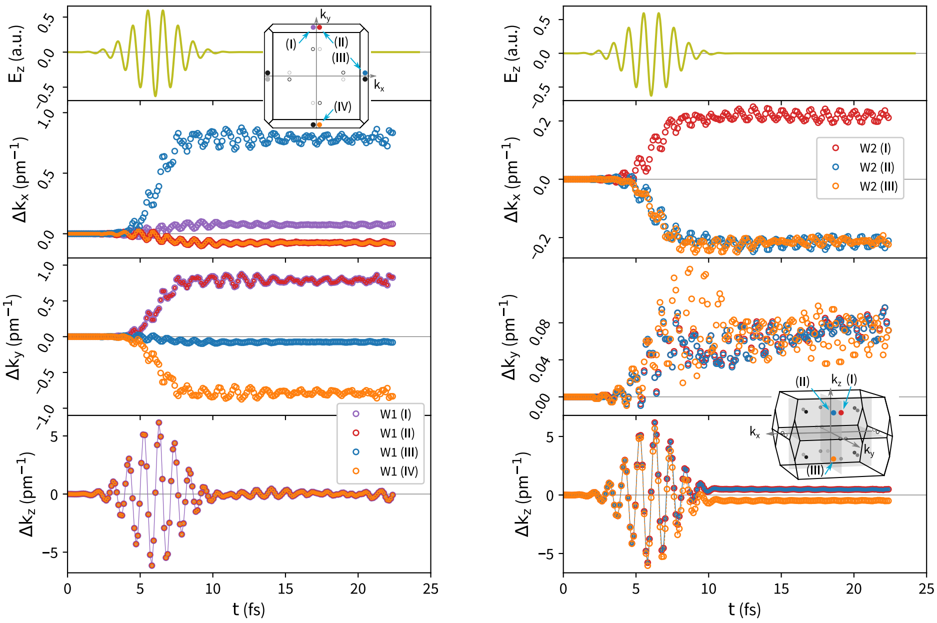

Fig. SI4 Comparison of time-dependent dynamics at different Weyl nodes. (left) W1 nodes, (right) W2 nodes. Laser pulse P_A is considered.

5 Inter Weyl node distance

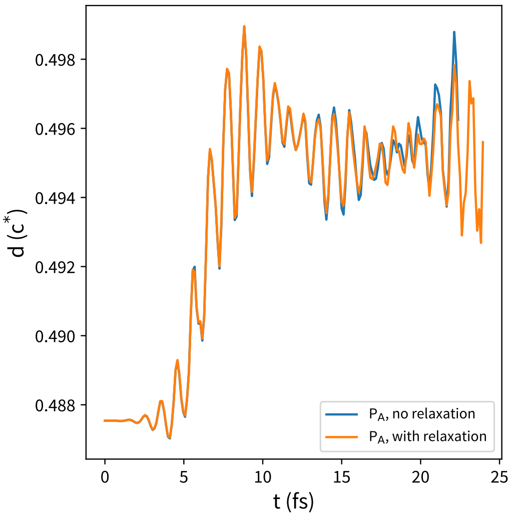

Fig. SI5 Weyl node separation in the k-space. Laser pulse induced change of the k-space distance between the nearest W1 and W2 Weyl nodes of different chirality. Both data for the evolution with and without the atomic site relaxation are depicted. Laser pulse P_A is considered.

6 Time-dependent current

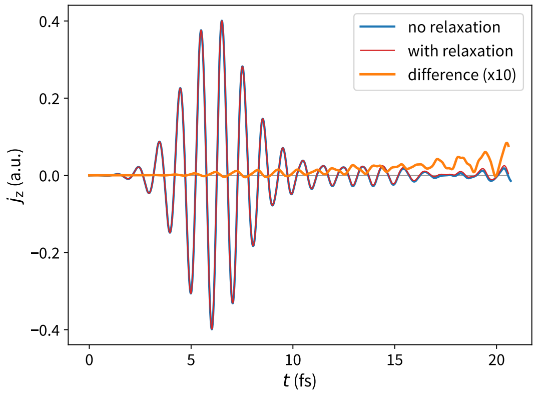

Fig. SI6 Time-dependent laser pulse induced total current. The j_z component parallel to the field is depicted. (red) current with atomic sites relaxation, (blue) current without relaxation , (orange) difference magnified by a factor of 10.

7 Lattice coupling induced modification of the WNs positions

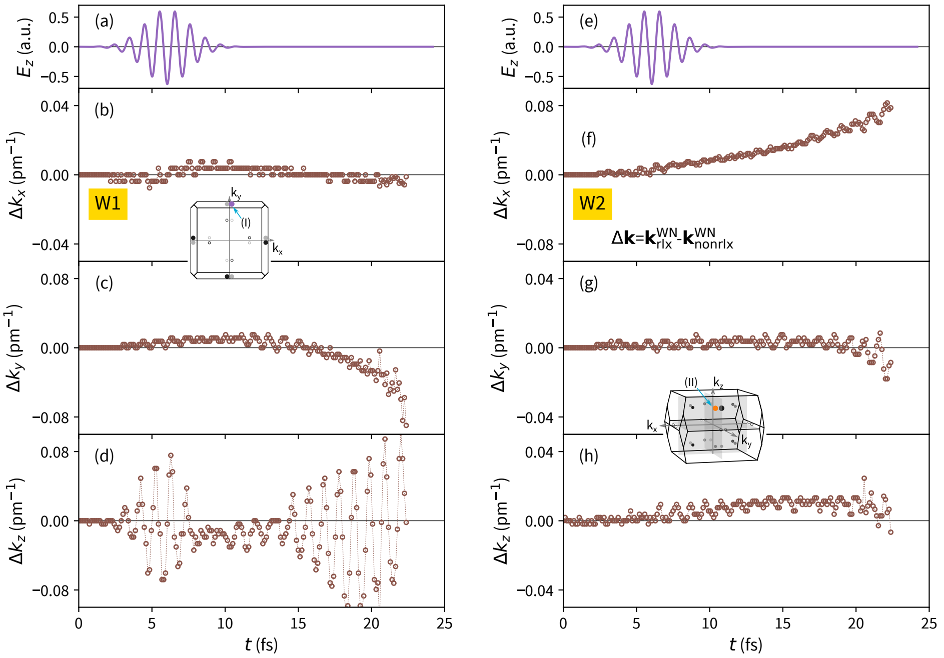

Fig. SI7 Influence of the lattice dynamics on the Weyl nodes' positions. Time-dependent modification of the Weyl nodes' positions induced by the inclusion of the Ehrenfest dynamics in TD-DFT calculations. The shown data are related to the WNs positions displayed in Figure 3. Cartesian axes are considered.

References

[1] Momma, K., Izumi, F.: *VESTA3* for three-dimensional visualization of crystal, volumetric and morphology data. Journal of Applied Crystallography **44**(6), 1272–1276 (2011) https://doi.org/10.1107/S0021889811038970
